# Identification of Closed Linear Epitopes in S1-RBD and S2-HR1/2 of SARS-CoV-2 Spike Protein Able to Induce Neutralizing Abs

**DOI:** 10.3390/vaccines11020287

**Published:** 2023-01-28

**Authors:** Yoshihiro Watanabe, Natsuko Hosokawa, Misaki Yoshida, Tomoyuki Miura, Mitsuhiro Kawano

**Affiliations:** 1 Innovative Clinical Research Center of Kanazawa University Hospital, Kanazawa 920-8641, Japan; 2 Rheumatology of Kanazawa University Hospital, Kanazawa 920-8641, Japan; 3 Institute for Life and Medical Sciences, Kyoto University, Kyoto 606-8507, Japan

**Keywords:** Ab-epitope, closed peptide epitope (CPE)-presenting antigen (Ag), SARS-CoV-2 spike, mutated epitope, universal epitope, sublingual vaccine, IgA

## Abstract

SARS-CoV-2 has evolved as several variants. Immunization to boost the Ab response to Spike antigens is effective, but similar vaccines could not enhance Ab efficacy enough. Effective Ab responses against the human ACE2 (hACE2)-mediated infection of the emerging SARS-CoV-2 variants are needed. We identified closed linear epitopes of the SARS-CoV-2 Spike molecule that induced neutralizing Abs (nAbs) against both S1-RBD, responsible for attachment to hACE2, and S2-HR1/2, in convalescents and vaccine recipients. They inhibited a pseudo-virus infection mediated by the hACE2 pathway. The epitope sequences included epitopes #7 (aa411-432), #11 (aa459-480) and #111 (aa1144-1161), in S1-RBD and S2-HR2. Epitope #111 was conserved in Wuhan and variant strains, whereas #7 and #11 were conserved in Wuhan carried mutations K417N and S477N/T478K in Omicron BA.4/5. These mutations were recognized by the original epitope-specific Abs. These epitopes in RBD and HR2 neither contained, nor overlapped with, those responsible for the antibody-dependent enhancement of the SARS-CoV-2 infection. The sublingual administration of multiple epitope-conjugated antigens increased the IgG and IgA Abs specific to the neutralizing epitopes in mice pre-immunized subcutaneously. The findings indicated that S1-RBD and S2-HR2 epitopes were responsible for pseudo-virus SARS-CoV-2 infections and that sublingual boosts with multiple epitope-conjugated antigens could enhance the protection by nAbs of IgG and IgA against infection by a wide range of variants.

## 1. Introduction

The first generation of vaccines against the severe, acute respiratory syndrome, coronavirus-2 (SARS-CoV-2), which targeted the viral Spike protein, was shown to prevent infection and control the severity of coronavirus disease 2019 (COVID-19). The large-scale, worldwide spread of SARS-CoV-2 in humans and the battle against the host immune responses resulted in the generation of viral mutations and the development of variant strains [1,2]. For example, the Omicron variant carries several mutations in the viral domain, binding to host hACE2, including the mutations N501, T478, L452, and K417, altering the affinity of the viral protein to hACE2 and greatly reducing the infection-blocking effects of the first-generation of SARS-CoV-2 vaccines and monoclonal Abs [3,4,5,6,7].

The ability of the virus to generate variant strains indicates the need to develop vaccines that express antigens able to induce Abs that bind to the epitopes encoded by these variants. Abs induced by these vaccines should therefore react with the immunizing antigen and be highly cross-reactive with the mutated Spike proteins present in the variant strains. Vaccination with the entire Spike protein possessing mutated RBD, however, would not efficiently induce mutated RBD-specific Abs, as most of these Spike protein-specific Abs would be unable to detect the few mutated amino acids of RBD [8,9,10,11,12,13,14]. The development of vaccines that generate Abs able to neutralize hACE2-mediated infection requires a consideration of the domain antigen of RBD and the defined epitope antigen [15,16,17,18,19]. Clinical trials have reported that several RBD-containing vaccines could induce nAbs and protect against SARS-CoV-2 infection with acceptable adverse events [10,20,21,22]. Moreover, conjugation to an appropriate, defined vaccine carrier or a formulation including adjuvants can be applied to a RBD domain and epitope vaccine to enhance the neutralization activity of RBD-specific Abs [11,12,13,20,21,22,23,24,25,26], and universal and/or cross-recognizing epitopes encoded by mutated variants could inhibit the binding of mutated RBDs to hACE2 [18,27,28,29,30]. In addition to RBD, the membrane fusion-critical regions that form the six helix bundles of S2-HR1/HR2 should be considered as target epitopes of vaccine antigens, as these regions were found to be rarely mutated in the known variants [2].

RBD has been reported to contain two linear neutralizing epitopes, aa440-460 and aa489-518, with conjugation of one or both of the epitopes to the carrier protein, inducing Abs that inhibit RBD binding to hACE2 [16,17,31]. Although both linear epitopes can be considered vaccine candidates able to induce nAbs, the region containing amino acids N501 to E516 has been classified as a group IV Ab-epitope associated with the Ab-dependent enhancement (ADE) of the infection [25,26]. As nAb and ADE epitopes can be distinguished by comparing group II/III and group IV epitopes, epitope-based vaccines should focus on nAb epitopes that do not contain ADE epitopes. Attempts using artificial intelligence have sought to determine whether immunogenic peptide sequences, including aa448-472, could act as B-cell epitopes, but these predicted epitope-specific Abs did not exhibit the neutralization activities shown by other epitope-specific Abs [14]. Although multiple linear epitopes displayed on a peptide array were found to react to Abs in the sera of patients convalescing from COVID-19, the sera of mice immunized with these linear epitopes did not significantly inhibit the pseudo-virus infection [16]. However, the induction of Abs specific to RBD epitopes facing the hACE2 molecule can inhibit the binding to hACE2-expressing cells well, as all SARS-CoV/CoV-2 variants, including the current variants, bind to hACE2 with similar affinity and all variant RBDs retain the same epitope areas [6,16,28]. The peptide-based vaccines have not shown enough prophylactic activity to date; therefore, an alternative strategy is needed to develop epitope peptide-based vaccines.

Closed peptide epitope-presenting antigens (CPE-Ags) can induce Abs that specifically recognize the native forms of proteins. The CPEs presented on antigen have several advantages, including the ability to efficiently generate Abs that recognize “inside” amino acid sequences, but not “blunt-end” neoepitopes, as well as the successful presentation of loop structures and sequence-dependent helical structures. Based on these advantages, CPE-Ags were utilized to generate Abs against several challenging antigens, such as the native form of linear poly-ubiquitination and different forms of hepcidin [32,33]. Moreover, these epitope-specific Abs have been shown to efficiently induce Abs that neutralize and/or regulate the functions mediated by the binding of the ligand and receptor. The present study describes the use of this CPE-Ag method to identify the Ab epitopes responsible for hACE2-binding and to detect the Ab-epitopes recognized by a sera of convalescents and vaccine recipients. The S1-RBD and S2-HR1/2 regions were evaluated to determine whether the selected individual epitope-specific Abs inhibited the pseudo-virus infection. Three epitopes were detected in these regions. Neutralizing epitope vaccines developed with CPE-Ag may have potential benefits when compared with the current SARS-CoV-2 vaccines.

## 2. Materials and Methods

### 2.1. Reagents and Peptide Synthesis

#### 2.1.1. Reagents

All of the chemicals were reagent grade. The HRP-conjugated goat anti-mouse IgG (H&L) (catalogue number: ab97023), anti-mouse IgA (ab97235), anti-human IgGs (qb6858), and anti-rabbit IgG (ab7090) were purchased from Abcam. The SARS-CoV-2 spike RBD recombinant protein (S1-RBD) (4-592-V08H) and S2 recombinant protein (40590-V08B) were purchased from Sino Biological. The CPE-bearing ovalbumin (OVA) antigens were characterized by SDS-polyacrylamide gel electrophoresis (SDS-PAGE) analysis, followed by Coomassie blue (BioRad, Hercules, USA. 1610803) staining (Supplementary Figure S1). The non-specific binding of mouse and human sera in ELISA was blocked using a solution containing 2% bovine serum albumin (BSA) (Sigma-Aldrich, St. Louis, MO, USA, 10735086001) and gelatin (Sigma-Aldrich, G7763).

#### 2.1.2. Peptide Synthesis

The entire amino acid sequence of WT SARS-CoV-2 RBD was covered by the set of 14 peptides. Individual peptides were designed as around 20-mer to 25-mer amino acids, with or without an appropriate linker sequence, i.e., G/S, GS or SG. Cysteine, in its reduced form, attached to N-terminal and C-terminal ends of each peptide lacking cysteine at its ends. These linear peptides were synthesized by solid phase synthesis using the Fmoc-method and separated by LC, and their molecular weights were analyzed by MS. Almost all of the peptides were >85% pure, whilst some were >70% pure. The sequences of the individual peptides are shown in Table 2, with described summary information. The regions of S2-HR1/2 are covered with 17 peptides, and their sequences are also shown in Table 2.

### 2.2. Preparation of CPE-Conjugated Antigens

#### 2.2.1. Conjugation of Linear Peptide on Maleimide OVA or KLH

The peptides were conjugated to a carrier protein, either OVA or KLH, using maleimide-containing linker-conjugated OVA or KLH (ThernoFisher Scientific, Waltham, MA, USA. 77125/77606). Each individual peptide was dissolved in PBS containing up to 20% DMSO, followed by the addition of three equivalents of each peptide to the solution of maleimide-activated OVA or KLH (1 mg/mL) and incubated in the refrigerator overnight. After the addition of cysteine-containing PBS at room temperature to quench the remaining excess maleimide, these solutions of individual peptide-conjugated OVA were used as CPE-Ags to induce epitope-specific Abs.

In addition, multiple peptides were conjugated to OVA or KLH by adding three equivalents of mixed peptides to the maleimide-activated protein, using the same procedure.

#### 2.2.2. SDS-PAGE Analysis of CPE-OVA

The molecular weights of the individual CPE-Ags conjugated to OVA were assessed by SDS-PAGE. The samples in the reduced buffer were heat denatured by incubation at 95 °C for 1 min, followed by electrophoresis on 5–20% polyacrylamide gels (ATTO), along with molecular markers. The gels were washed, fixed and stained with Coomassie Brilliant Blue. The stained bands, which are higher molecular weight with several kDa than OVA band, were observed in the individual peptide-conjugated antigens (supplementary Figure S1).

### 2.3. Immunization via Subcutaneous (SC) and Sublingual (SL) Routes

#### 2.3.1. SC Immunization

Solutions containing individual CPE-Ags (10 μg) plus 100 μg Quil A (Sigma-Aldrich, St. Louis, MO, USA. S4521) in 100 μL PBS were injected SC into the backs of the mice at 2 weeks intervals. If CPE-specific IgG Abs were not generated, the mice were injected SC with a newly prepared antigen using the same procedure.

#### 2.3.2. SL Administration

The experimental mice were anesthetized with isofuran, followed by the SL administration of 3 μL(low dose) or 10 μL (high dose) of a 1 mg/mL solution of mixed CPEs-Ags; as a control, some mice were administered with the vehicle alone. The solution was administered three times per week for two or four weeks.

### 2.4. ELISA for Mouse Sera, Human Sera, and Other Purified Rabbit Abs

#### 2.4.1. Detection of IgG Abs-Specific to Individual Epitopes and Proteins

Venous blood samples were obtained from the mice immunized with individual CPE-Ags, followed by centrifugation at 3000 rpm (Sakuma, SL-IVD). The resulting serum samples were stored at −30 °C before use.

CPE-specific IgG Abs were analyzed using mixed CPEs conjugated to KLH. Briefly, 100 μL of 1.0 mg/mL solution CPEs conjugated to KLH in PBS were added to each well of a 96-well plate, and the plates were incubated overnight at 4 °C. The plates were washed thoroughly and 200 μL of blocking buffer, consisting of a mixture of 2% BSA and gelatin in PBS at 4 °C. The plates were washed four times with 0.05% Tween containing PBS (PBST), followed by the addition of the serum samples containing mice, human or rabbit Abs diluted appropriately in blocking buffer. The plates were incubated for 1 h at room temperature or overnight at 4 °C, washed four times with PBST, incubated for 30 min at room temperature with a 1:10,000 dilution of the appropriate secondary Abs. The plates were again washed four times with PBST, followed by the addition of a solution containing the enzymatic substrate 3,3′,5,5′-tetramethylbenzidine (TMB). After incubation for 15 to 30 min, the stop solution (0.2 N H_2_SO_4_) was added and the absorbance of the wells at 450 nm was assessed using a microplate reader (BioRad, iMark).

To analyze the cross-reactivity of the Abs towards mutant epitopes of the Omicron and Delta strains, peptides containing mutated sequences (K417N, L452R or T478K) were conjugated to KLH and added to 96-well plates, as described above. The plates were subsequently incubated with mouse antisera specific to the wild-type #7, #10 and #11 CPEs, and the cross-reactivity was assessed using the above procedure.

To analyze the ability of individual CPE-specific antisera to recognize RBD and S2 proteins, each well of a 96-well plate was coated with 100 μL of a 1.0 μg/mL of solution of the respective protein. After incubation with the blocking buffer, 1:1000 dilutions of individual CPE-specific mouse sera were added, and the binding was assessed, as described above.

#### 2.4.2. Epitope-Mapping of Human Sera and Purchased Rabbit Polyclonal Abs Using Individual CPEAgs

To identify the common epitopes recognized by convalescents from COVID-19 and individuals immunized with the mRNA vaccine, these sera were collected after obtaining approval from the ethics committee for our institution, and stored at −30 °C before use. Wells of the ELISA plate were coated with individual CPE-Ags, consisting of 14 RBD peptides and 17 HR1/2 peptides conjugated to OVA, followed by incubation with the blocking buffer. The subsequent procedure was identical to that described in Section 2.4.1, with the exception that the secondary Ab consisted of HRP-conjugated goat anti-human IgG Abs.

The positive controls consisted of two polyclonal rabbit Abs specific to RBD or S2. Individual wells of microtiter plates were coated with 31 individual CPE antigens, as above, followed by incubation with the appropriate dilutions (1:10,000 to 100,000) of these polyclonal Abs. The ELISA procedure was identical to that in Section 2.4.1, with the exception that the secondary Abs consisted of HRP-conjugated goat anti-rabbit IgG Abs.

### 2.5. Binding Assays of RBD and ACE2

Biotinylated hACE2 was synthesized by incubating a 1:1 molar ratio of succinyl propionyl biotin (Dojin Chemical, Kumamoto, Japan, 346-06351) and hACE2 protein (SinoBiologicals, Wayne, NJ, USA. 10108H08H) on ice, overnight and at room temperature, for 1 h. A 50 μL aliquot containing 100 ng RBD protein was added to each well of a 96-well microtiter plate (NUNC, 44-2404-21), followed by overnight incubation at 4 °C. The plates were blocked by incubation with 3% BSA in PBS, followed by incubation with an appropriate dilution of biotinylated hACE2 on ice for 30 min. After washing with PBST, HRP-conjugated streptavidin was added to each well, and the plate was incubated for 30 min. The plate was washed four times with PBST, followed by the addition of the TMB substrate solution. The enzymatic reaction was stopped by the addition of 0.2 N H_2_SO_4_, and the absorbance of each well at 450 nm was measured with a colorimetric microplate reader (BioRad).

### 2.6. Neutralization of Pseudo-Type Lentivirus Infection

A 100 µL aliquot of medium containing hACE2-expressing CRFK cells at a concentration of 2.5 × 10^5^ cells/mL was added to each well of a 96-well plate 1 day before infection. Previously harvested samples containing pseudo-typed lentiviral particles coated with the SARS-CoV-2 S protein were thawed, serially diluted two-fold for a total of nine times and triplicate aliquots of these dilutions were transferred to the wells of the 96-well plate. The control wells contained the medium alone, with no virus. For infection, a 100 µL aliquot of each LpVspike(+) or LpVspike2(+) dilution was added to each well containing hACE2-expressing CRFK cells, and the plates were incubated at 37 °C for 2 days in an atmosphere containing 5% CO_2_. The luciferase activity of these CRFK cells was measured by adding 50 µL of cell lysis solution (Toyo B-Net) to each well, followed by agitation for 15 min. A 30 µL aliquot of each lysate was transferred to a well of a Nunc F96 MicroWell white plate (ThermoFisher Scientific, Waltham, USA), followed by the addition of 30 µL of luminescent substrate. The luciferase activity was measured using a TriStar LB 941 reader (Berthold Technologies, Bad Wildbad, Germany) and the MikroWin software. The ID50 values were calculated as previously described [34]

Neutralization assays were performed using adjusted viral doses that yielded equivalent infectivity levels for each cell line. The relative infection rate (%) was represented as compared to the RLU readings measured in the virus control wells (cells plus virus without test sample) after subtracting the background RLU values from the cell control wells (cells only, without virus or test sample). Pseudo-typed viruses were added to a final concentration of 6000 RLU mL^−1^ to the ACE2-expressing CRFK cells. The cells were incubated at 37 °C for 48 h in an atmosphere containing 5% CO2, and then the luciferase activity of the samples was measured.

### 2.7. Statistical Analyses

The statistical difference regarding the OD values of each group of the sublingual administration experiment were statistically compared through a Student *t* test.

## 3. Results

### 3.1. Induction of S1 RBD Epitope-Specificantisera Inhibiting the Binding of RBD to hACE2

#### 3.1.1. Induction of S1-RBD Epitope-Specific IgG Abs in Mice

A total of 14 individual peptides were conjugated with OVA-maleimide to form a CPE-Ag. Two mice in each epitope were set and immunized with individual CPE-Ag, two to four times at two weeks intervals. Then, the ability of the sera from these immunized mice to recognize the epitopes was tested by the multiple epitope-conjugated KLH, as described in M and M.

**Figure 1:**
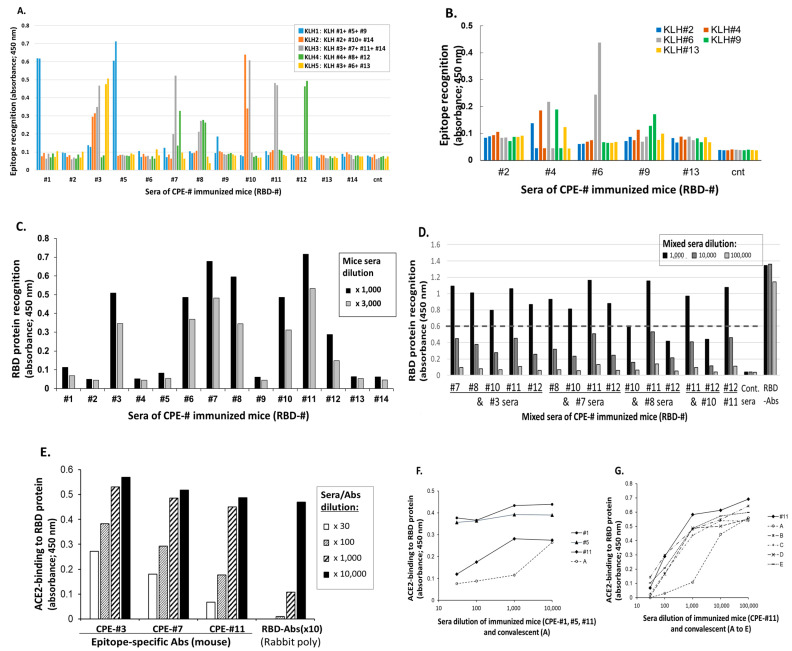
Selection of epitopes in S1-RBD in which specific antisera are functional. (**A**) Induction of CPE-specific IgG Abs in mice immunized with individual CPE-Ags covering RBD amino acid sequences (experiment 1). The sera of two mice immunized with individual CPE-Ag were evaluated for recognition of CPEs presented on irrelevant carrier protein KLH. The sera dilution was x1000-fold, and positive signals were defined as s/n>5 compared to the signals of sera of non-immunized mice. The OD values showed means of duplicate assays in diluted sera of each two mice. (**B**) Confirmation of CPE-specific IgG Abs induction in two other mice immunized with individual S1-RBD CPE-Ags (experiment 2). Epitopes #2, #4, #6, #9 and #13-specific Abs were performed again for confirming the experiment 1. (**C**) Assessment of RBD protein recognition in all CPE-specific antisera. Individual CPE-specific IgG Abs were examined for recognition of recombinant S1-RBD protein. The serial dilutions of mice sera in each CPE group were assessed for binding to S1-RBD recombinant protein. (**D**) Additive recognition by combinations of each single epitope-specific antisera. The individual #3, #7, #8, #10, #11 or #12 epitope-specific antisera was combined with each other and assessed if equal mixtures of them enhanced signals of RBD recognition. (**E**) Identification of RBD-epitopes recognizing antisera able to inhibit binding of ACE2. The #3, #7 and #11 CPE-specific antisera inhibited binding of RBD and hACE2 proteins, and commercially available anti-RBD polyclonal Abs (purified) inhibited this binding effectively. The OD values shown as bar graph are means of triplicate assays. (**F**,**G**) Comparison of inhibitory activities against RBD-hACE2 binding in RBD-recognizing epitope-specific antisera, RBD-unrecognizing epitope-specific antisera and moderate/severe COVID-19 patients’ sera were evaluated by RBD-hACE2 binding assays. As control mice sera, the epitope #1 and #5-specific antisera unable to recognize RBD protein were also set up in parallel, and these two sera did not inhibit the binding of RBD to hACE2 protein.

Almost all of the CPE-Ags were found to induce the IgG Abs specific to the individual epitopes (Figure 1A,B). For example, the sera of the individual mice immunized with CPE peptide #1, #5 and #9 could recognize the KLH bearing these three peptides. Using this method, most of these amino acid sequences, with the exception of aa336-342, aa361-379 and C-terminal aa500-525, were covered by the set of individual CPE-specific IgG Abs generated in the mice immunized with CPE-Ags. Although experiment 1 failed to detect the CPE #6-specific IgG Abs (Figure 1A), experiment 2 found that the sera of two mice were able to recognize this peptide-presenting KLH (KHL#6; Figure 1B).

#### 3.1.2. RBD Protein Recognition by CPE-Specific Abs

The analyses of the individual Abs specific to CPEs showed that seven of these Abs recognized RBD protein (Figure 1C), although some of these CPE-specific Abs could not recognize the protein. For example, the Abs specific to CPE #1 and CPE #5 had high titers to these peptides, but could not recognize RBD protein.

The regions detecting RBD were localized to three areas, CPE #3, CPEs #6 to #8, and CPEs #10 to #12. The examination of the combinations of these epitope-specific Abs showed that the combination of the Abs specific to CPEs #7 and #11 could effectively enhance the RBD recognition of either Ab (Figure 1D).

#### 3.1.3. Ability of CPE-Specific Antisera to Inhibit Binding to hACE2

The ability of the individual CPE-specific antisera to recognize RBD protein was assessed by analyzing the ability of each peptide to inhibit the binding of RBD to hACE2 protein. The mouse antisera specific to CPEs #3, #7 and #11 were found to inhibit the binding of RBD to biotin-hACE2 protein in a concentration-specific manner (Figure 1E). Hundred-fold and greater dilutions of antisera specific to CPEs #11 and #7 were found to inhibit hACE2-binding by ~50%.

To assess the neutralizing activities of these CPE-specific antisera, the antisera specific to CPE #11 were compared with the sera of patients convalescing from COVID-19 infection, including one patient, conv-A, who was convalescing from severe COVID-19 with a prolonged high SARS-CoV-2 viral load, and four other patients, conv-B to conv-E, who were convalescing from less severe COVID-19. The inhibitory activity of the conv-A serum was more than 10-fold higher than the inhibitory activities of the sera from the mice immunized with CPE #11 (Figure 1F,G). In contrast, the inhibitory activities of the conv-B to conv-E sera were similar to that of the mice with CPE #11-specific Abs (Figure 1G). As the negative controls, the sera of the mice immunized with CPEs #1 and #5, which did not recognize RBD protein (Figure 1C), could not inhibit hACE2 binding to RBD protein, even at 30-fold dilution (Figure 1F).

Taken together, these findings indicate that certain single epitope-specific Abs can block SARS-CoV-2 infection mediated by binding to hACE2. The Abs in the mice immunized with specific epitopes could inhibit RBD-hACE2 binding to an extent that is comparable to that of the sera in patients convalescing from moderate COVID-19. This selection identified three neutralizing CPEs—#3, #7 and #11—that could inhibit hACE-2 binding to RBD protein.

### 3.2. Induction of S2-HR1/2 Epitope-Specific Antisera Able to Recognize S2 Protein

#### 3.2.1. Induction of S2-HR1/2 Epitope-Specific IgG Abs in Mice Using CPE Antigens

**Figure 2:**
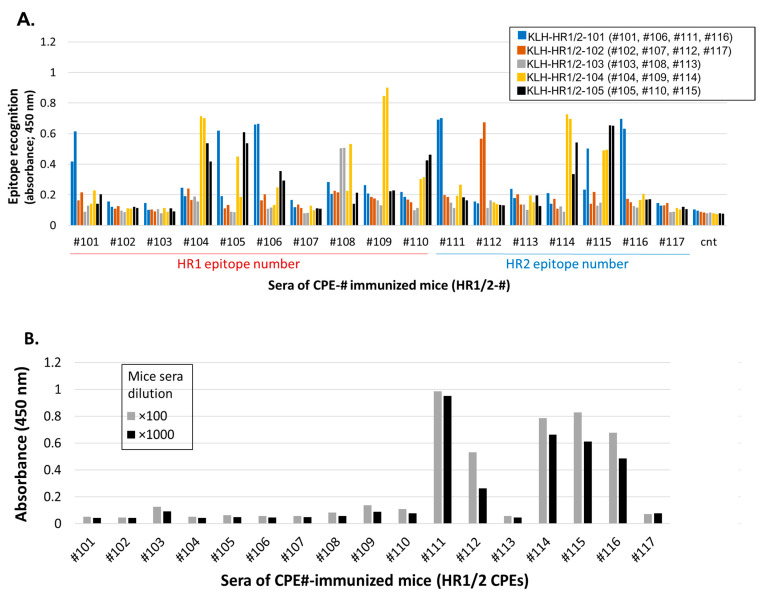
Selection of epitopes in S2-HR1/2 regions in which specific antisera are functional. (**A**) Induction of CPE-specific Antisera in mice immunized with individual S2-HR1/2 CPE-antigens (first trial). The sera of two mice immunized with individual CPE-Ag were evaluated for recognition to CPEs presented on irrelevant carrier protein KLH. The sera dilution was x1000-fold, and positive signals were defined as s/n > 5 compared to the signals of sera of non-immunized mice. (**B**) Assessment of recombinant S2 protein recognition in each CPE-specific Abs. Individual CPE-recognizing Abs were examined for their recognition to recombinant S2 protein. The serial dilutions of mice sera were assessed for binding to S2 protein.

The antisera specific to S2-HR1/2 were generated by the same process as described above, using the set of CPE-Ags covering the HR1/HR2 regions. These CPE-Ags of S2-HR1/2 were found to induce individual CPE-specific IgG Abs (Figure 2A). The use of multiple CPEs bearing KLH showed that CPE-specific IgG Abs covering a wide range of the HR1 and HR2 regions were generated in the mice by immunization, with corresponding CPE-conjugated OVA. The levels of IgG Abs specific to CPEs derived from HR1, such as CPEs #104, #105, #108, #109 and #110, were compared with those of the IgG Abs specific to CPEs derived from HR2, such as CPEs #111, #112, and #114 to #116.

Almost the entire S2-HR1/2 regions were found to be covered by epitope-specific IgG Abs, with the exception of short sequences of aa921-928, aa971-976, aa1169-1173 and C-terminal aa1214-1223.

#### 3.2.2. S2 Protein Recognition by CPE-Specific Antisera

The abilities of these CPE-specific IgG Abs of the S2-HR1/2 regions to recognize S2 protein were tested. Interestingly, only antisera, specific to the CPEs of the HR2 region, could recognize S2 protein, whereas antisera specific to the CPEs of the HR1 region could not, or could only slightly, recognize S2 protein. These results were identical with the results showing that commercially available rabbit Abs specific to S2 protein did recognize the CPEs derived from HR2, but did not recognize any CPEs of HR1 (supplementary Figure S3). Figure S3 also indicated that the immunization of S2 protein to obtain polyclonal Abs in rabbits did not induce Abs-recognizing HR1 region epitopes.

These results suggest that the epitopes of the S2-HR2 region were displayed on the surface of the protein and were immunologically recognized, but that the HR1 region epitopes were not displayed on the surface, thus preventing B cell recognition, as well as the access of Abs.

#### 3.2.3. Identification of Common Epitopes in S2-HR1/2 Using CPE-Ags

This panel of OVA presenting individual CPEs was used to examine the ability of the sera from both the convalescents and vaccine recipients to detect the IgG Abs specific to the S2-HR1/2 regions. These sera recognized CPEs localized within the HR2 region, but did not recognize, or recognized less of, the epitopes (#101 to #110) covering the HR1 region.

Representative epitope profiling of the sera from two convalescents showed that CPEs #111 and #114/115 were two of the major epitopes recognized by these sera (supplementary Figure S4; upper). These sera also weakly recognized CPEs #104/#105 in the HR1 region, but did not detect any major epitopes in this region. CPE #111 was recognized by four of the five sera, with these sera having high IgG Ab titers (Table 1 and Figure S4). Taken together, these findings show that the common epitopes recognized by the convalescents’ sera were similar to those recognized using a peptide array format [19,25,26].

The serum samples of the Spike mRNA-vaccinated recipients recognized the same HR2 epitopes as the convalescent sera, with CPEs #111 and #114/115 being two of the major epitopes identified by the sera from the vaccinated individuals. The representative epitope-profiling of five participants is shown in the supplementary Figure S4 (lower). The evaluation of the epitopes recognized by the sera from convalescents and vaccine recipients, and their frequencies, showed that CPEs #111 and #114/#115 were major epitopes and CPE #104/#105 was a minor epitope (Table 1).

These findings strongly suggest that the HR1 region is hidden inside the HR2 helical structure and cannot induce specific Abs. The inability to induce Abs specific to the peptides of the HR1 region (Figure 2B, Table 1, supplementary Figure S4) was not due to the lack of immunogenicity, but was likely due to a structural hindrance, resulting in the lack of immunological presentation of this region. The absence of IgG Abs specific to the epitopes in the HR1 region in the sera from convalescents and vaccine recipients and from commercially available S2-specific Abs (supplementary Figure S3) was most likely due to the lack of immune recognition and selection.

### 3.3. Antisera Recognizing Defined CPEs Inhibit Pseudo-Virus Infection in a Manner Mediated hACE2

#### 3.3.1. Pseudo-Virus Infection of CRFK Cells via SARS-CoV-2 Spike Molecule

**Figure 3:**
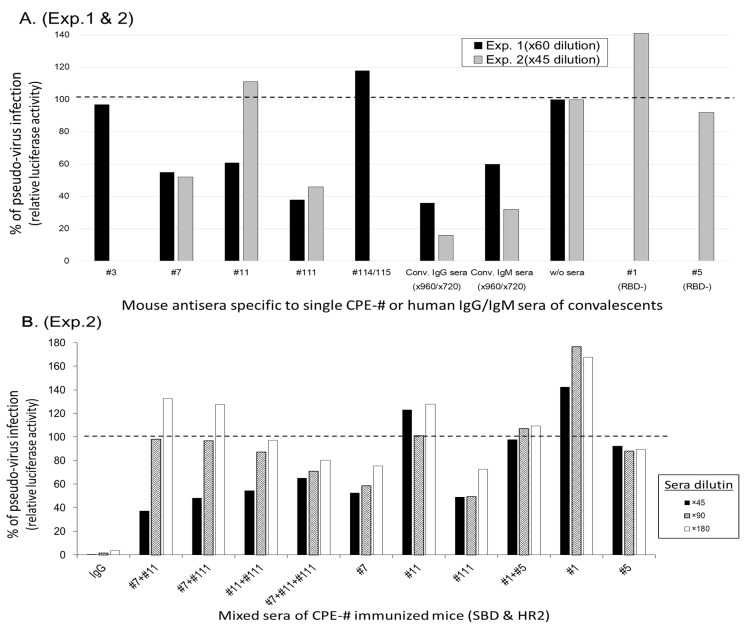
Protective ability of epitope-specific antisera in SARS-CoV-2 pseudo-virus infection. (**A**) Pseudo-virus infection and inhibition by mouse sera immunized with multi CPEs-Ag. In exp. 1 (black bars), mouse sera at 60-fold dilution were assessed in the pseudo-virus infection system of SARS-CoV-2 Spike molecule. The #7, #11 and #111 CPEs-specific antisera but not #3 and #114/#115 CPEs-specific antisera inhibited this pseudo-virus infection. The exp. 2 (gray bars) was conducted with 45-fold dilution. As controls, convalescents’ sera were set in parallel at 960-fold dilution (black bars) in exp. 1 and 720-fold dilution (gray bars) in exp.2. (**B**) The sera dilution-dependent inhibition of pseudo-virus infection. In exp. 2, two or three sera specific to individual neutralizing epitopes (#7, #11 and #111) or non-neutralizing epitopes (#1 and #5) were mixed equally, then the mixed sera were diluted to culture media and added at final dilutions of 45-, 90- and 180-fold to see inhibition against the pseudo-virus infection.

Candidates of the CPE-specific antisera were selected, and they were assessed to inhibit the pseudo-virus infection of hACE2-expressing CRFK cells, according to the assay system established [34]. As shown in Figure 3A (exp. 1; black bars), two kinds of sera (IgG-major and IgM-major) of the convalescents recovering from severe COVID-19 inhibited around 50% of the pseudo-virus infection at dilutions of up to 960-fold. In comparison, 60-fold dilutions of murine antisera, specific to CPEs #7, #11 and #111 CPE-Abs, were observed compared to the 50% inhibition of pseudo-virus infection, and the CPE #3 and #114/#115 antisera did not inhibit pseudo-infection. To confirm the activities of the murine antisera against CPEs #7, #11 and #111, these epitope-specific Abs were tested both singly and in combination. As shown in Figure 3A (exp. 2; gray bars), each single epitope-specific antisera (#7 and #111) reduced the pseudo-virus infection by 50%. In contrast, the murine antisera specific to CPEs #1 and #5, which are unable to recognize RBD protein, did not inhibit the pseudo-virus infection. The IgG and IgM from the sera of patients convalescing from severe COVID-19 inhibited pseudo-virus infection by over 50% at a 720-fold dilution, and almost completely inhibited pseudo-virus infection at a 90-fold dilution. In exp. 2, the combinations of the antisera specific to CPE #7, #11 and #111 were assessed. As shown in Figure 3B, the dilution-dependent inhibitions were observed in the cases of the antisera combinations of CPE #7, #11 and #111, but not in case of the antisera combination of CPE #1 and #5.

These findings show that the Abs directed against CPEs #7, #11 and #111, both singly and in combination, inhibited pseudo-virus infection in an additive, but not a synergistic, manner; however, these inhibitory activities were over 10-times weaker than those of the IgG and IgM from patients convalescing from severe COVID-19.

#### 3.3.2. Selection of Ab-Epitopes Inhibiting the Process of SARS-CoV-2 Infection

Table 2 summarizes the process from the generation of epitope-specific IgG Abs to the identification of protective Ab-epitopes against pseudo-virus infection.

**Table 2:** Summary of process to identify Ab-epitopes protective SARS-CoV-2 pseudo-virus infection.

			Stepwise Selection of Infection-Responsible Epitopes of SARS-CoV-2 Spike *			
Epitope #	Amino Acid Number	Amino Acid Sequence of SARS-CoV-2 Spike	Generation of Epitope-Abs ^(1)^	Protein Recognition of Epitope-Abs ^(2)^	Epitope Characterization	Pseudovirus Infection ^(5)^
RBD					Inhibition of ACE2-binding by epitope-Abs ^(3)^	
1	316-336	SNFRVQPTESIVRFPNITNLC	+	−		
2	336-355	CPFGEVFNATRFASVYAWNR	−	−		
3	343-361	NATRFASVYAWNRKRISNC	+	+	+	−
4	361-379	CVADYSVLYNSASFSTFKC	−	−		
5	380-392	YGVSPTKLNDLCF	+	−		
6	391-414	CFTNVYADSFVIRGDEVRQIAPGQ	+	+	−	
7	411-432	APGQTGKIADYNYKLPDDFTGC	+	+	+	+
8	406-427	EVRQIAPGQTGKIADYNYKLPD	+	+	−	
9	431-448	GCVIAWNSNNLDSKVGGN	−	−		
10	446-467	GGNYNYLYRLFRKSNLKPFERD	+	+	−	
11	459-480	SNLKPFERDISTEIYQAGSTPC	+	+	+	+
12	479-499	PCNGVEGFNCYFPLQSYGFQP	+	+	−	
13	500-515	TNGVGYQPYRVVVLSF	−	−		
14	504-525	GYQPYRVVVLSFELLHAPATVC	−	−		
S2-HR1					Recognition by convalescent sera ^(4)^	
101	901-920	QMAYRFNGIGVTQNVLYENQ	+	−	−	
102	911-929	VTQNVLYENQKLIANQFNS	−	−	−	
103	920-940	QKLIANQFNSAIGKIQDSLSS	−	−	−	
104	929-950	SAIGKIQDSLSSTASALGKLQD	+	−	+/−	
105	943-964	SALGKLQDVVNQNAQALNTLVK	+	−	+/−	
106	950-971	DVVNQNAQALNTLVKQLSSNFG	+	−	−	
107	963-985	VKQLSSNFGAISSVLNDILSRLD	+	−	−	
108	976-995	VLNDILSRLDKVEAEVQIDR	+	−	−	
109	988-1006	EAEVQIDRLITGRLQSLQT	+	−	−	
110	999-1019	GRLQSLQTYVTQQLIRAAEIR	+	−	−	
S2-HR2						
111	1144-1161	ELDSFKEELDKYFKNHTS	+	+	+	+
112	1154-1168	KYFKNHTSPDVDLGD	+	+	−	
113	1163-1181	DVDLGDISGINASVVNIQK	−	−	−	
114	1174-1193	ASVVNIQKEIDRLNEVAKNL	+	+	+	−
115	1184-1202	DRLNEVAKNLNESLIDLQE	+	+	+	−
116	1197-1213	LIDLQELGKYEQYIKWP	+	+	−	
117	1205-1223	KYEQYIKWPWYIWLGFIAG	−	−	−	

* Criteria for positive (+) and negative (−) per assay and evaluation. (1) Positive (+) signals towards epitope-conjugated KLH were set over s/n > 5 at 1000-fold diluted mouse sera, compared to the background signals to KLH without any epitope peptides. Negative (−) signals were below the positive criteria. (2) Positive signals were set over s/n > 5 at ×1000-fold diluted mouse sera in RBD or S2 protein-coated plate, compared to the background and towards non-coated wells. (3) Positive signals were set around 50% inhibition and more at ×100-fold mouse diluted sera, compared to control sera. (4) Positive (+) was defined as major recognition in individual human sera of convalescent and vaccinated volunteers, intermittent (+/−) was minor recognition in individual samples, and negative (−) was below s/n < 5 at ×1000-fold diluted sera. (5) Positive signals were set around 50% inhibition and more at ×60-fold or ×45-fold diluted mouse sera compared to pseudo-virus infection without sera or with irrelevant epitope-specific IgG containing sera.

We have identified three CPEs #7, #11 and #111 as the nAb-epitopes from both the RBD and HR1/2 of the SARS-CoV-2 Spike molecule. These epitopes-specific antisera were able to recognize recombinant protein, to inhibit the binding of RBD and hACE2, and to inhibit pseudo-virus infection. The identified Ab-epitopes were also recognized by the sera from patients convalescing from SARS-CoV-2 infection and people vaccinated with Spike mRNA vaccines.

### 3.4. SL Administration of Defined CPEs-Ag Induces IgA Abs Specific to nAb-Epitopes

#### 3.4.1. Experimental Setting of SL Administration to Assess Its Boosting Ability

**Figure 4:**
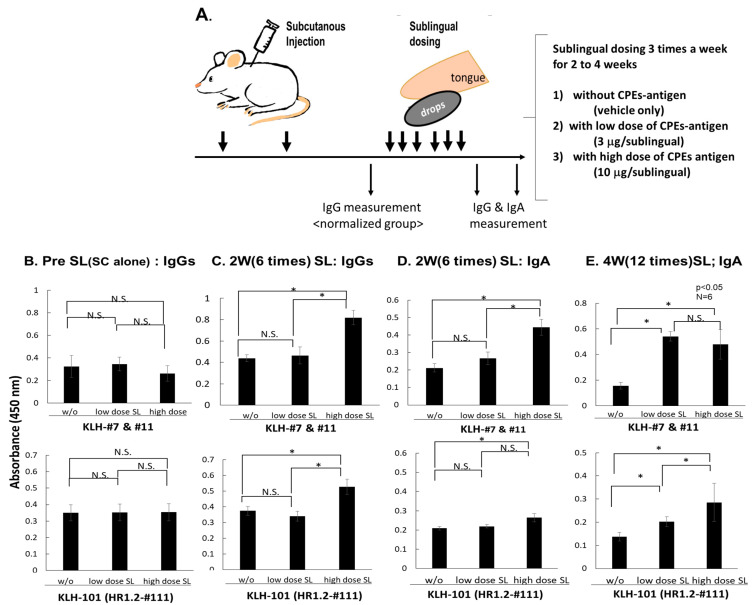
Enhancement and induction of IgG and IgA Abs-specific to nAb-epitopes by SL boosting of CPE-Ag. (**A**) Experimental scheme of SL dosing after 2 times SC administration to assess if Abs-specific to these CPE-Ags can be enhanced. The experimental design is shown. Mice were immunized by SC route with CPE #7 + #11 + #111-conjugated antigens (CPEs-OVA) at the dose of 3 μg or 10 μg/mice and administered again 2 weeks later. (**B**) Further 2 weeks after 2nd dose of SC immunization, mice were divided into three groups with similar IgG titers specific to CPE #7 + #11-conjugated KLH and CPE #111-conjugated KLH, such that the mean IgG values of the three groups were not significantly different each other. The SL drop solution was applied under the tongue and stood for 1 min. This SL administration was repeated 3 times a week for 2 weeks and 4 weeks. The Ab-recognition towards CPE #7, #11 and #111 were shown before (**B**) and 2 weeks (**C**,**D**) after starting SL boosting in mice immunized with mixed CPEs-antigens via SC route. (**E**) IgA-responses were further analyzed after 4-weeks with SL dosing. N.S. means not significant each other, and asterisk (*) means statistically significant each other with *p*-value <0.05.

Vaccination with CPE-Ags is expected to specifically enhance the generation of nAbs without increasing other Abs, including non-nAbs and those related to ADE. As the IgA Abs specific to nAb-epitopes could play a critical role in SARS-CoV-2 infection, methods have been developed to enhance the generation of IgA Abs that specifically recognize nAb-epitopes. Thus, the ability of the SL administration of CPEs-Ag to induce IgA, as well as IgG, Abs was tested (Figure 4A). Mice were subcutaneously immunized twice with CPEs-Ag, consisting of two RBD-derived CPEs #7 and #11, and one HR2-derived CPE #111. Subsequently, six mice in each group were further administered via the SL route with a low dose (3 μg) or high dose (10 μg) of CPEs-Ag, or with the vehicle, thrice a week. As shown in Figure 4B, in each group, there was no significant difference in terms of the IgG values recognizing CPE #7+#11 (upper graph) and CPE #111 (lower graph).

#### 3.4.2. Enhanced Production of IgG and IgA Specific to Neutralizing Epitopes by SL Administration

The CPE-specific IgG Abs were significantly increased in the mice administered a high-dose (10 μg) SL antigen three times per week for two weeks (Figure 4C). Moreover, the IgA Abs specific to these CPE-Ags were also generated in this group (Figure 4D). Both CPE-specific IgG and IgA titers were also increased in the mice administered a low dose (3 μg) SL antigen thrice a week for four weeks (Figure 4E).

## 4. Discussion

The present study describes the identification of the Spike protein epitopes of the SARS-CoV-2 variants that are able to generate antisera that inhibit RBD binding to ACE2 and pseudo-virus infection via the hACE2 pathway. IgG Abs specific to single epitopes were generated by immunizing mice with individual CPE-Ags, with two of these epitopes—#7 (aa410-430) and #11 (aa460-480)—in the RBD of Spike protein, generating an antisera able to inhibit hACE2-binding. A third conserved epitope, #111 (aa1140-1160), in the S2-HR2 region of the Spike molecule, induced an antisera that was able to recognize S2 protein and inhibited pseudo-virus infection, similar to the sera of convalescent patients. These findings indicate that this CPE-Ag method is useful for inducing functioning Abs that recognize the native forms of Spike protein.

**Figure 5:**
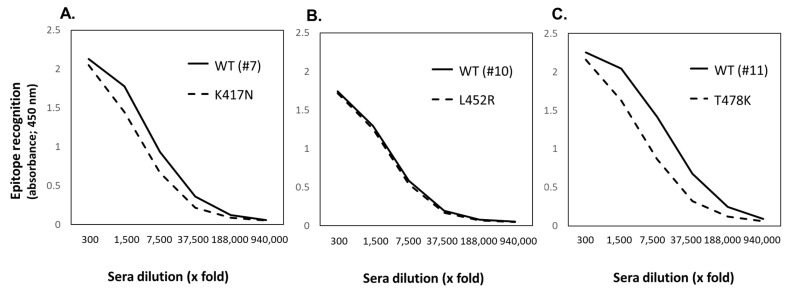
Cross-recognition of Wuhan strain epitope-specific Abs towards mutated epitopes of Omicron/Delta strain (**A**–**C**). The antisera specific to CPE #7 (**A**), #10 (**B**) and #11 (**C**) epitopes were assessed for cross-recognition towards mutation-inserted epitopes (dotted lines) and corresponding original epitopes (solid lines), respectively. The mutated epitopes synthesized by Fmoc-chemistry were conjugated on maleimide-activated KLH. Then both original CPE epitope and mutated CPE-conjugated KLH were individually coated on the microtiter plate as described in Materials and Methods section.

Epitopes #7 and #11 are mutated in several variants of SARS-CoV-2, including the Omicron and Delta variants, whereas epitope #111 is conserved in the Wuhan strain and all known variants. In Figure 5, it is shown how the individual CPE-specific mouse antisera can recognize mutated epitopes as CPE-Ages of Delta/Omicron. The mutant sequence of epitope #7 (K417N) showed similar dose responses, with a slight decrease, to the IgG Abs specific to the original CPE #7-Ag. In addition, the mutant epitope #11 (T478K) decreased the recognition of the original CPE #11-specific Abs by around 3- to 4-fold, with a parallel dose-response curve. In the case of the mutant epitope #10, characteristic of the Delta variant, the original CPE #10-specific Abs equally recognized. These findings strongly suggest that antigens bearing mutated epitopes can be recognized by Wuhan-type epitope-specific Abs and that the original Abs-specificities could adapt to mutant epitope-specificity.

Omicron variants of SARS-CoV-2 have been found to contain around ten mutation sites in the RBD. Seven of these sites are characterized by five CPEs-Ags, including CPEs #7, #10, #11 and #12. Antigens containing these mutant sequences can likely be used to evaluate the cross-reactivity of the existing Ab-responses in participants.

This epitope-level analysis of neutralization can be applied to other functions of Abs. For example, ADE has been observed in the sera of individuals convalescing from SARS-CoV and SARS-CoV-2 infection [35,36]. Ab-dependent cellular cytotoxicity (ADCC) mediated by S antigen-specific Abs has been reported as another risk of excess humoral immunity in SARS-CoV-2 infection [37]. Three-dimensional crystal structure analysis, using recombinant Abs from these patients, revealed the localization of the epitopes of Abs possessing ADE activity to the RBD, with some overlapping to the neutralizing epitopes [37,38]. Patients with severe COVID-19 were reported to have a high ratio of Abs possessing ADE activity, with the linear epitope peptides shown to inhibit the ADE activity of these patients’ sera [39]. Importantly, the Abs possessing ADE activity have been categorized as type IV neutralizing Abs and could affect the up-down conformation of RBD in Spike proteins [40]. In cases of ADCC risk, this epitope-level analysis may allow us to develop a much safer vaccine.

The epitope-level analyses (Table 1, supplementary Figures S2–S4) clearly indicate that polyclonal Abs recognizing the entire Spike molecule include, not only nAbs, but Abs with ADE Abs, Ab-dependent cellular cytotoxicity (ADCC) and other risk-related Abs [36,37,38,39]. The findings in this study and those previously reported suggest that the neutralization vs. non-neutralization activities in induced Abs could be epitope-dependent. The epitopes identified in this study were not identical and they did not overlap with the previously identified ADE epitopes [38,39,40]. Several ADE-epitopes have been identified outside or near the RBM of RBD, but not the ACE2-facing regions [36,39], which may be one reason that the nAb-epitopes identified in this study do not overlap to the reported ADE-epitopes. In the case of the S2 region, it has not been fully clarified in terms of ADE risk yet, but it is unlikely that ADE epitopes exist in the hydrophobic six-helix bundle region because this region is critical to cell-fusion via direct insertion into the lipid bilayers of cell membranes.

The present study identified the Spike protein epitopes involved in RBD-hACE2 binding by utilizing CPE-Ags that were able to generate specific IgG and IgA Abs. CPE-Ags were found useful for identifying functional Ab-generating epitopes and can be useful as vaccine antigens to efficiently induce and enhance nAbs (Figure 1, Figure 2 and Figure 4). Abs induced by linear B-cell epitopes have been shown to inhibit pseudoviral infections [31,36]. The present study clearly demonstrates the usefulness and superiority of inducing nAbs against hACE2-binding RBD epitopes when these epitopes are presented as CPE-Ags.

Panels of single CPE-Ags can also reveal epitope-specific Ab profiles, thereby revealing the breadth and intensity of antigen-specific Abs (supplementary Figure S4). The neutralizing CPE #111 and the non-neutralizing CPE #114/115 are two of the major Ab-generating epitopes in the S2-HR1/HR2 regions (Table 1). These epitopes are widely recognized by the sera of convalescents and vaccine recipients with different Ab titers. The Ab profiles at the epitope level are useful for assessing the relationship between each patient’s Ab profile and the ability to inhibit viral infection. For example, the presence of a high Ab titer against CPE #111, but a low Ab titer against CPE #114/115, might suggest that the individual has high inhibitory activity against all viral variants, as the significant inhibition of pseudo-virus infection was only observed in the sera specific to #111 epitope, but not to #114/115 epitope (Figure 3). In cases of recipients lacking #111 epitope-specific Abs, the inhibitory activities against pseudo-virus infection may be lower than the recipients possessing #111-specific Abs.

Although Abs recognizing “discontinuous” and “conformational” epitopes have been reported to be overlooked by linear peptides, the CPE method described in this study can present the loop and helical structures formed by the presented linear peptides. Thus, “continuous” and “conformational” epitopes can be displayed as CPEs on scaffold proteins, despite the inability of the CPE method to cover “discontinuous” epitopes. For loop structures acting as epitopes, the CPE method can avoid the generation of blunt-end neoepitopes and present loop structures derived from the characteristics of their sequences. Peptides consisting of 20~25 amino acids have been shown to be suitable for the formation of loop structures [32,33] and our unpublished observations.

Two epitopes of RBD, predicted to form a loop structure, were found to be responsible for the attachment and binding to the hACE2 molecule (Figure 1 and Figure 3). Epitopes in the S2-HR2 region, consisting of CPE #111 and CPE #114/#115, were shown to localize to the stem helix and the six-helix bundle with a high helical structure [41,42]. Recent studies using peptide arrays also found that the Abs in the sera of convalescents frequently recognized epitopes in the HR2 region, but not those in the HR1 region [17,23,31,35]. Abs specific to linear epitopes in the sera of mice immunized with peptide antigens, however, did not inhibit the infection of the pseudo-virus, suggesting that many neo-epitope-specific Abs recognize the blunt-ends of peptides, but did not recognize Spike protein efficiently.

In contrast to linear arrays, the CPE method allows the presentation of loop and helical structures, avoiding the creation of blunt-end neoepitopes. This results mainly in the induction of Abs that recognize native proteins. Abs induced by CPE antigens can therefore bind to “inside” amino acid sequences of target molecules and can recognize loops and helical structures. Although patients’ sera can be monitored by arrays of linear peptides, CPE-Ages more effectively induce Abs that recognize native forms of proteins than linear peptides.

RBD-ACE2 binding was inhibited to a similar extent by murine CPE-specific Abs and by the sera of patients convalescing from moderate COVID-19 (Figure 1F,G), and a boost of CPE-Ags can increase specific IgG and IgA Abs in mice (Figure 1, Figure 2 and Figure 4). The pseudo-virus-neutralizing titers of these antisera were over ten times lower than those of the sera of patients convalescing from severe COVID-19 (Figure 3). Combinations with the appropriate adjuvants and alternative administration methods and routes could enhance specific Ab titers and class-switching, toward Ig classes and IgG subclasses that could more effectively prevent SARS-CoV-2 infection [26,43,44]. In fact, the authors reported that IgA class monoclonal Abs (mAbs) are much more effective to IgG class mAbs [34].

The present study also found that the SL administration of antigen could enhance IgG Ab titers and induce class switching to IgA. In Figure 4, class switching of IgG to IgA was observed to occur after the SL administration of 10 µg antigen thrice weekly for two weeks. Furthermore, IgG and IgA titers were increased after the SL administration of 3 µg antigen thrice weekly for four weeks, suggesting that the IgA Abs specific to nAb-epitopes can be induced and enhanced by repeated SL administration. These findings suggest that the induction of epitope-specific IgA Abs could enhance the inhibition of pseudo-virus infection. The IgA mAbs showed greater inhibition of pseudo-virus infection than the same Fab-bearing IgG mAbs [34]. As the main action of the Abs specific to neutralizing epitopes is the protection against initial infection, both the increase of IgG titers and the induction of mucosal IgA Abs are important, as secreted IgA plays a major role in mucosal immunity, and SARS-CoV-2 infection occurs primarily in mucosal organs, such as the lungs.

mRNA vaccines have been reported to induce IgA Ab secretion at titers greater than the IgA titers in the sera and the mucosal organs of patients convalescing from COVID-19. However, the mechanisms of the induction of IgA through the intramuscular injection of mRNA-LNP vaccines have not been clarified [45]. In addition to being enhanced by the SL administration of CPE-Ags, the secretion of IgG and IgA class nAbs by mucosal organs may be enhanced by the use of the appropriate antigens. Although the currently available mRNA vaccines induce IgA Abs, the maintenance of IgA titers is considered critical for inhibiting primary infections. In addition to SL sensitization, further SL administration concomitant with appropriate adjuvants should be assessed if these schedules can enhance IgA titers to a level higher than those of the current mRNA vaccines.

The advantages of vaccination using CPE-Ag vaccination may include the specific enhancement of nAbs without increasing other Abs, such as non-neutralizing Abs and Abs-related to ADE. The SL administration of multiple CPE-Ag possessing variant sequences can only induce nAb-epitope-specific Abs, as well as inducing the Ig-class to switch toward secretory IgA Abs. The specificity of these induced Abs could be tuned to recognize variant epitopes derived from the original Wuhan strain. These advantages of CPE-antigen vaccines suggest that this SL vaccination is an alternative way to enhance the nAbs that are able to prevent the primary infection of the emerging variants of SARS-CoV-2.

## 5. Conclusions

The CPE-Ag method enabled the identification of three epitopes located in S1-RBD or S2-HR2. One was a universal epitope, present in all known variants of SARS-CoV-2, with the other two being epitopes mutated in the Omicron variants. Abs induced by the epitope of the Wuhan strain can cross-recognize the epitopes of the Omicron variants. The SL administration of these CPE-Ags for two-to-four weeks can also enhance IgG titers and induce class switching to IgA of the Abs specific to these epitopes. These advantages of CPE-Ag vaccines suggest that this SL vaccination is well suited to the existing SARS-CoV-2 vaccines to prevent the infection of variant viruses.

## 6. Patents

All data are included in the PCT patent applied.

## Figures and Tables

**Table 1:** Common epitopes in S2-HR1/2 region recognized by convalescents’ sera and sera of recipients vaccinated twice with Spike mRNA.

Participant\CPE Number	CPE # of Abs Positive * (Strong Positive)		
	#104/#105	#111	#114/#115
Convalescents recovered from moderate and severe COVID-19 (out of five convalescents)	3/5 (0/5)	4/5 (3/5)	3/5 (2/5)
Participants vaccinated with Spike mRNA-LNPs (out of ten particiants)	2/10 (0/10)	8/10 (6/10)	7/10 (4/10)

* Positive criteria are over >5 of s/n ratio.

## Data Availability

Data are contained within the article and supplementary information.

## Supplementary Materials

The following supporting information can be downloaded at: https://www.mdpi.com/article/10.3390/vaccines11020287/s1, Figure S1. SDS-PAGE analysis of CPE-presenting OVA and an image of CPEs-Ag. Figure S2. Recognition of RBD CPE-antigens by RBD-specific polyclonal Abs. Figure S3. Recognition of S2-HR1/2 CPE-antigens by S2-specific polyclonal Abs. Figure S4. Epitope-mapping of convalescents’ sera and sera of vaccinated participants.
