# Supplementary material for: Identification of Closed Linear Epitopes in S1-RBD and S2-HR1/2 of SARS-CoV-2 Spike Protein Able to Induce Neutralizing Abs
Source: Vaccines (Basel). 2023 Jan 28;11(2):287. doi: 10.3390/vaccines11020287 (PMC9966687; doi:10.3390/vaccines11020287)
Supplement: Supplementary file 1 [file vaccines-11-00287-s001.zip › vaccines-2095621-supplementary.pdf]

Supplementary information (Vaccines-2095621)

1. Design of peptide-epitope and preparation of closed peptide epitope (CPE)-antigen.

The whole amino acid sequences of WT RBD of SARS-CoV-2 (Wuhan strain) were covered by the set of 14 peptides of #1 to #14, as shown in Table 2. Individual peptides were designed as around 20-mer to 25-mer amino acids without or with appropriate linker sequence G/S, GS or SG. Then, Cysteine as reducing form sulfhydryl group was added at N-terminal and C-terminal end in the case of lacking Cystein at N/C-terminal ends, in order to cyclize on carrier protein as the closed peptide epitope (CPE).

In supplementary Figure S1, several kDa upper bands of original ovalbumin (OVA) were shown by staining using Coummasie blue dye. The right SDS-PAGE gel showed bands of #114, #115 or #116 epitope-conjugated OVA, and left PAGE gel showed the bands of #116 or #117 epitope-conjugated OVA. The conjugation of #117 might be lower yield than other epitopes-conjugation, since the MW of #117-conjugated OVA is slightly lower than those of #116 and other epitope-conjugated OVA. These analyses showed that the dimer formation with double MW (ca.90-100 kDa) and higher MW did not occur or less occur as observed same as minor bands existed in original OVA. The bottom cartoon is an image of CPE-presenting antigen, which peptide epitopes are conjugated as forms of loop structure and helical structure on scaffold protein.

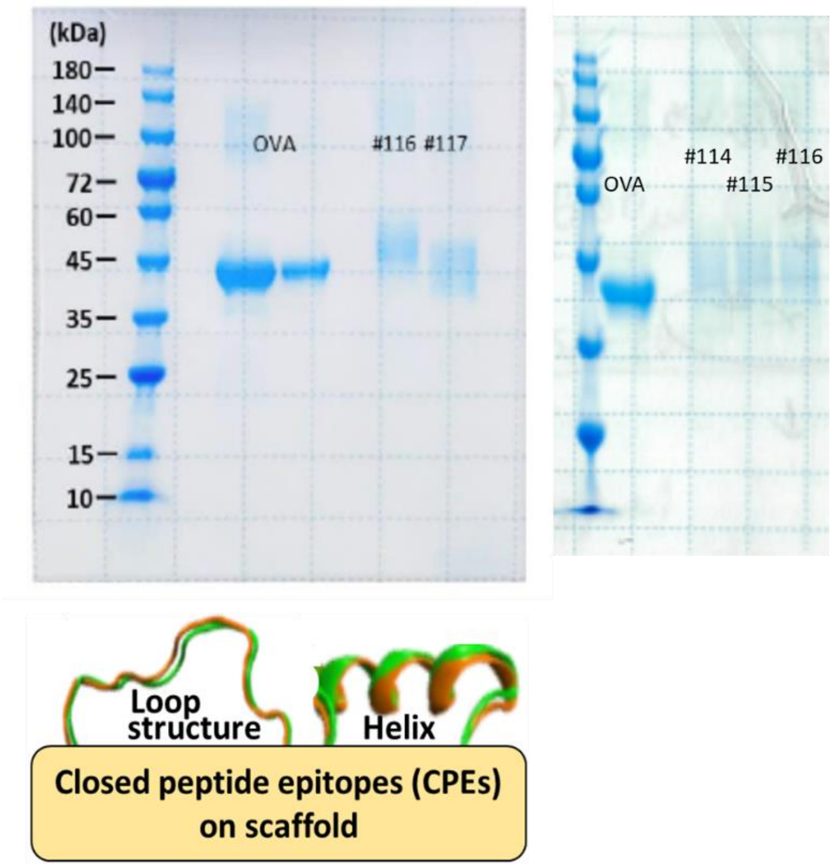

Supplementary Figure S1. SDS-PAGE analysis of CPE-presenting OVA and an image of CPEs-Ag.

2. Evaluation of polyclonal Abs specific to RBD or S2 protein by the CPE-OVA sets.

To evaluate the individual CPE-OVA in terms of immunological recognition, the commercially available RBD-specific polyclonal Abs and the S2-specific polyclonal Abs were assessed if the individual CPE-OVA can be recognized by them.

The results shown in supplementary Figure S2 and S3 indicated that nine to eleven out of 14 CPE-OVA covering RBD can be recognized by polyclonal Abs-specific to RBD protein, and that almost of all CPE-OVA of S2-HR2 region but not S2-HR1 region CPE-OVA can be detected by S2-specific Abs. The details of these results are discussed in the Discussion of the paper, but it was revealed at least that the CPE-antigen detected by these polyclonal Abs are definitively presented similar as epitopes of the RBD and S2 protein.

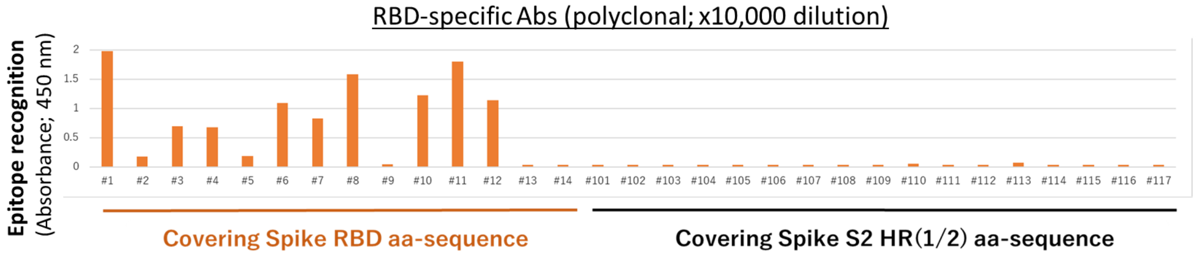

Supplementary Figure S2. Recognition of RBD CPE-OVA by RBD-specific polyclonal Abs

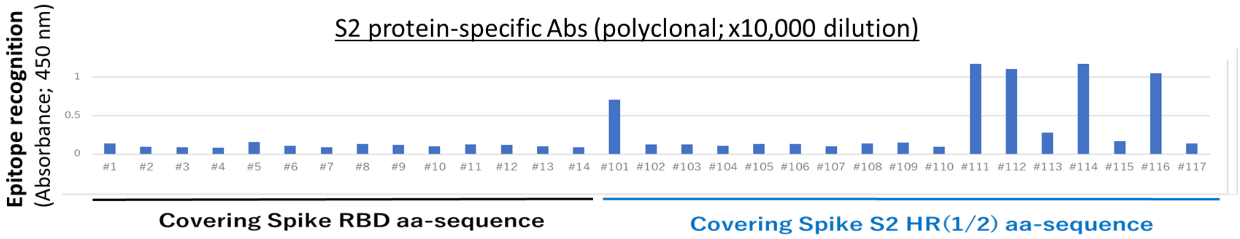

Supplementary Figure S3. Recognition of S2-HR1/2 CPE-OVA by S2-specific polyclonal Abs

3. S2-HR1/2 epitope-mapping of convalescents' sera and sera of participants vaccinated with Spike mRNA-LNPs using the CPE-OVA set.

The panel of individual CPE-presenting OVA covering S2-HR1 and HR2 regions was used to examine the recognition of sera from both convalescents and vaccine recipients at 1,000-fold dilution. These sera recognized CPEs localized within the HR2 region including the stem helix (#111 to #117), but did not recognize any of the epitopes (#101 to #110) in the HR1 region.

In supplementary Figure S4 (upper), the representative epitope profiling of sera from two convalescents showed that CPEs #111 and #114/115 were two of the major epitopes recognized by these sera. These sera also weakly recognized CPEs #104/#105 in the HR1 region, but did not detect any major epitopes in this region. It was also shown in Figure S4 (lower) that serum samples of Spike mRNA-vaccinated recipients recognized the same HR2 epitopes, with CPEs #111 and #114/115 being two of the major epitopes as identified by convalescents' sera.

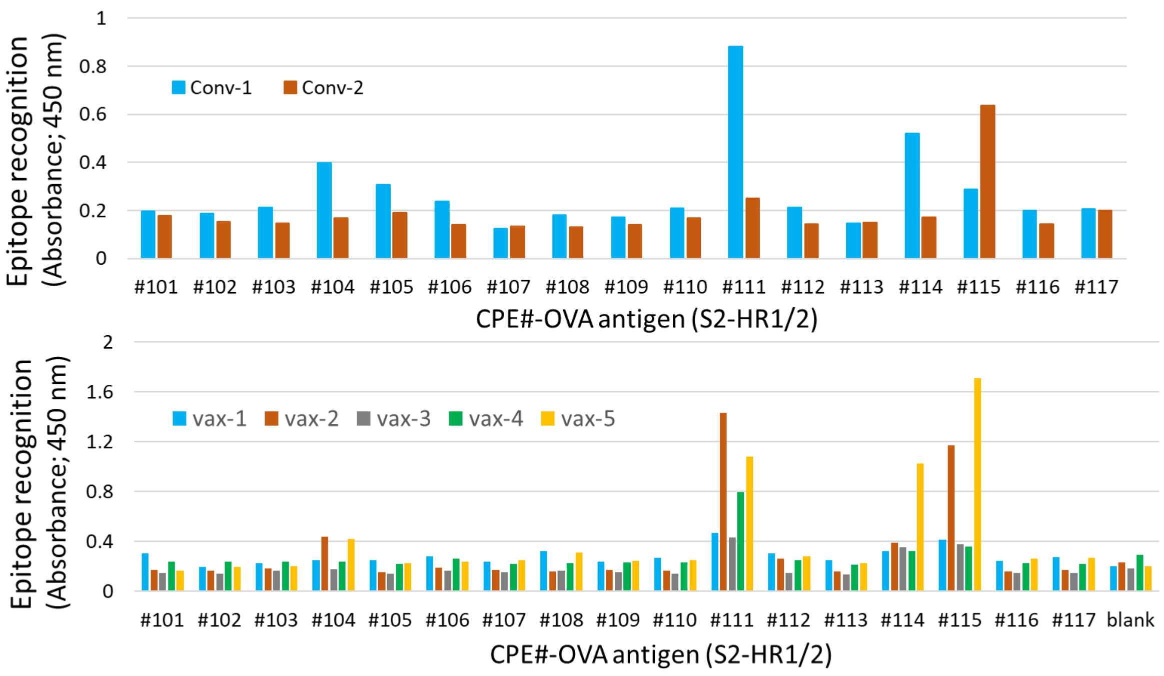

Supplementary Figure S4. Epitope-mapping of convalescents' sera and sera of vaccinated participants.
